# Pharmacologic concentrations of linezolid modify oxidative phosphorylation function and adipocyte secretome

**DOI:** 10.1016/j.redox.2017.05.026

**Published:** 2017-05-31

**Authors:** Laura Llobet, M. Pilar Bayona-Bafaluy, David Pacheu-Grau, Elena Torres-Pérez, José M. Arbones-Mainar, M. Ángeles Navarro, Covadonga Gómez-Díaz, Julio Montoya, Ester López-Gallardo, Eduardo Ruiz-Pesini

**Affiliations:** a Departamento de Bioquímica, Biología Molecular y Celular, Universidad de Zaragoza, C/ Miguel Servet 177, 50013 Zaragoza, Spain; b Instituto de Investigación Sanitaria de Aragón (IIS Aragón), Universidad de Zaragoza, C/ Miguel Servet 177, 50013 Zaragoza, Spain; c Centro de Investigaciones Biomédicas en Red de Enfermedades Raras (CIBERER), Universidad de Zaragoza, C/ Miguel Servet 177, 50013 Zaragoza, Spain; d Department of Cellular Biochemistry, University Medical Center, Humboldtalle 23, 37073 Göttingen, Germany; e Unidad de Investigación Traslacional, Instituto Aragones de Ciencias de la Salud (IACS), Hospital Universitario Miguel Servet, Paseo de Isabel la Católica 1-3, 50009 Zaragoza, Spain; f Centro de Investigaciones Biomédicas en Red Fisiopatología de la Obesidad y Nutrición (CIBERObn), Hospital Universitario Miguel Servet, Paseo de Isabel la Católica 1-3, 50009 Zaragoza, Spain; g Servicio de Otorrinolaringología, Hospital Universitario Miguel Servet, Paseo de Isabel la Católica 1-3, 50009 Zaragoza, Spain; h Fundación ARAID, Universidad de Zaragoza, C/ Miguel Servet 177, 50013 Zaragoza, Spain

**Keywords:** Linezolid, Oxidative phosphorylation, Adipocyte, Secretome, Apolipoprotein E, Adipokine

## Abstract

The oxidative phosphorylation system is important for adipocyte differentiation. Therefore, xenobiotics inhibitors of the oxidative phosphorylation system could affect adipocyte differentiation and adipokine secretion. As adipokines impact the overall health status, these xenobiotics may have wide effects on human health. Some of these xenobiotics are widely used therapeutic drugs, such as ribosomal antibiotics. Because of its similarity to the bacterial one, mitochondrial translation system is an off-target for these compounds. To study the influence of the ribosomal antibiotic linezolid on adipokine production, we analyzed its effects on adipocyte secretome. Linezolid, at therapeutic concentrations, modifies the levels of apolipoprotein E and several adipokines and proteins related with the extracellular matrix. This antibiotic also alters the global methylation status of human adipose tissue-derived stem cells and, therefore, its effects are not limited to the exposure period. Besides their consequences on other tissues, xenobiotics acting on the adipocyte oxidative phosphorylation system alter apolipoprotein E and adipokine production, secondarily contributing to their systemic effects.

## Highlights

• Linezolid decreases oxidative phosphorylation system capacity.
• Linezolid reduces adipocyte differentiation from human adipose-derived stem cells.
• Linezolid modifies APOE, adipokine and extracellular matrix proteins levels.
• Linezolid changes DNA methylation of human adipose tissue-derived stem cells.
• Xenobiotics, acting on adipocyte oxidative phosphorylation, affect human health.

## Graphical abstract

**Figure:**
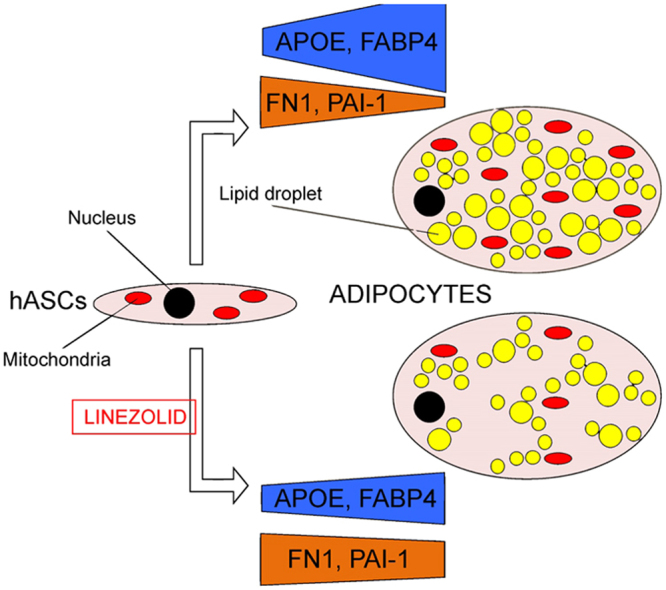

## 1 Introduction

The oxidative phosphorylation system (OXPHOS) includes the electron transport chain (ETC) respiratory complexes I to IV (CI-CIV) and the ATP synthase (complex V, CV). Several OXPHOS subunits are encoded in the mitochondrial DNA (mtDNA). This molecule also codes for 2 ribosomal (rRNAs) and 22 transfer (tRNAs) RNAs needed for the expression of the 13 mtDNA-encoded polypeptides. Mature cells require OXPHOS function to perform many of their activities but it is also important for cell differentiation.

Genetic manipulation of the OXPHOS system affects adipocyte differentiation. This manipulation appears to be accompanied by a defect in adipokine secretion. Thus, it has been reported that a mutated thymidine kinase 2 (TK2) knock-in mouse showed mtDNA depletion in white adipose tissue and reduced fat accumulation. There was also a severe reduction in leptin mRNA and circulating protein levels [1]. Mouse 3T3-L1 cells knocked down for the mitochondrial transcription factor A (TFAM) showed a decrease in mtDNA copy number, levels of ETC subunits, CI and CIV activities, and oxygen consumption. These cells also showed a diminished adiponectin expression at both gene and secreted protein levels [2]. Adiponectin mRNA expression was also decreased in TFAM knocked down human mesenchymal stem cells (hMSCs) [3]. Mouse 3T3-L1 or adipose tissue derived stem (mASCs) cells that lacked the CR6/gadd45-interacting protein Gadd45gip1/Crif1, a translation/assembly factor for mtDNA-encoded polypeptides, expressed lower levels of mtDNA-encoded subunits and displayed disrupted adipocyte differentiation, accompanied by a reduced adiponectin expression [4].

Physiologic manipulation of OXPHOS can also affect adipokine secretion. Oxygen is the ultimate electron acceptor in the ETC and hypoxia restrained adipogenic differentiation in both mouse preadipocytes and hMSCs [3], [5]. It has been found that adiponectin and leptin release was increased and decreased, respectively, in human adipocytes differentiated at 10% oxygen compared with 21% [6].

We have previously shown that some OXPHOS xenobiotics could alter the adipocyte differentiation of human adipose tissue-derived stem cells (hASCs) [7]. In addition to genetic and physiologic intervention, chemical manipulation of OXPHOS can also affect adipokine secretion. It was found that CI inhibitor rotenone decreased adiponectin secretion of rat adipocytes and adiponectin mRNA expression in hMSCs [3], [8]. Capsaicin, another CI inhibitor, decreased leptin and increased adiponectin expression in 3T3-L1 adipocytes [9]. The CIII inhibitor antimycin A reduced adiponectin mRNA levels in mouse 3T3-L1 cells [10]. The CV inhibitor oligomycin diminished adiponectin mRNA levels and secreted adiponectin in mouse 3T3-L1 cells [2], [10].

Some OXPHOS xenobiotics are widely used therapeutic drugs. For example, nucleoside reverse transcriptase inhibitors (NRTIs) are used against the human immunodeficiency virus (HIV), the agent causing the acquired immune deficiency syndrome (AIDS). However, these drugs can also inhibit the DNA polymerase gamma (POLG), the enzyme required for mtDNA replication. Thus, OXPHOS function was reported as a common target of NRTI toxicity [11]. NRTIs reduced adipocyte differentiation and leptin secretion by hASCs-derived adipocytes [7]. They also decreased adiponectin mRNA expression and protein secretion in mouse 3T3-L1 and 3T3-F442A cells and in primary human subcutaneous preadipocytes [12], [13], [14]. Systemic adiponectin levels were also reduced in patients under antiretroviral therapy [15]. The ribosomal antibiotic linezolid (LIN) is considered a promising option in the treatment of multidrug resistant tuberculosis [16]. Unfortunately, side effects, such as myelosuppression, lactic acidosis and optical and peripheral neuropathy have been associated to this antibiotic [16]. At concentrations below the steady-state peak serum concentration [17], LIN inhibited hASCs mitochondrial protein synthesis and decreased the triglycerides (TGs) amount and secreted leptin in hASCs-derived adipocytes [7].

Adipokines modulate many metabolic pathways and have a broad range of systemic actions [18]. The previous observations indicated that an OXPHOS blockage modifies the secretion of some adipokines. Then, an adipocyte OXPHOS disruption caused by off-target effects of therapeutic drugs might affect people health status. Therefore, we analyzed the effect of LIN on adipocyte secretome to uncover factors whose secretion was altered.

## 2 Materials and methods

### 2.1 Chemicals

All reagents used were of research or cell culture quality. Chloramphenicol (CAM) and LIN were purchased from Sigma-Aldrich (St Louis, MO, USA).

### 2.2 Cells, growth and differentiation conditions

StemPro® Human Adipose-Derived Stem Cells (#R7788-115, Gibco™, Thermo Fischer Scientific) derive from human adipose tissue collected during liposuction procedures and were cryopreserved at passage 1 from primary cultures. Each lot of hASCs originates from a single donor of human lipoaspirate tissue, and the two previously characterized lots used here [7], are named hASCs-1 and hASCs-2. In some experiments, we used hASCs derived from 13 different donors, after a signed informed consent was obtained [19].

These cells were generally grown in MesenPRO RS™ medium (Gibco™, Thermo Fischer Scientific), containing 5 mM glucose and 2% fetal bovine serum (FBS) complemented with MesenPRO RS™ growth supplement and 2 mM L-glutamine prior to use. To avoid undesired phenotypic effects, cells were grown in the absence of antibiotics [20].

To induce adipogenic differentiation, confluent hASCs were incubated for 21 days with StemPro® Adipogenesis Differentiation Kit (Gibco™), as previously described [7].

### 2.3 Analysis of adipocyte differentiation

Intracellular lipids were stained with the hydrophilic stain Nile Red that, when partitioned in a hydrophobic environment, becomes fluorescent. For quantitative determination of Nile Red fluorescence, the NovoStar MBG Labtech microplate instrument was used (Ex: 485 nm / Em: 572 nm).

Adipogenesis Detection Kit (Abcam) was used to quantify TGs accumulation in cells according to the manufacturer's instructions. In this assay, TGs are solubilized and hydrolyzed to glycerol, which is subsequently oxidized to convert the probe to generate color (λ_max_ = 570 nm). A NovoStar MBG Labtech microplate instrument was used for measurements.

For quantitative determination of adiponectin and leptin in cell culture supernatants, Human Adiponectin ELISA kit (Millipore) and Leptin Human ELISA Kit (Abcam) were used [7], according to the manufacturer's instructions.

For immunocytochemistry, the cultured cells were fixed with 4% paraformaldehyde for 15 min at room temperature and permeabilized with 0.1% Triton X-100 for 10 min. After blocking with 0.1% bovine serum albumin (BSA), the washed cells were incubated for 1 h at room temperature with a primary antibody against APOE (Abcam). Subsequently, the cells were incubated with fluorescence-labeled secondary Alexa Fluor® 594 (Molecular Probes) at room temperature for 30 min, protected from light. The cells were further incubated with 1 μM 4′,6-diamidino-2-phenylindole (DAPI) for nuclear staining. Between incubations, samples were washed with phosphate buffered saline (PBS) containing 0.05% Tween.

### 2.4 Analysis of mitochondrial function

The enzymatic activity of citrate synthase (CS) was assayed following previously described protocols [21]. Protein concentration was determined by the Bradford protocol (#500-0006; Bio-Rad) [22]. CIV activity and levels were determined using the Complex IV Human Specific Activity Microplate Assay Kit (Mitosciences, Abcam®) according to the manufacturer's instructions.

### 2.5 Genetics characterization and gene expression analysis

*APOE* genotyping was performed by polymerase chain reaction (PCR) amplification and sequencing using primers and conditions described elsewhere [23], [24]. These sequences were obtained using the BigDye Terminator v3.1 Cycle Sequencing Kit (Applera Rockville, MD, USA) and an ABI Prism 3730xl DNA analyzer (Applied Biosystems, Foster City, CA, USA).

For quantitative determination of the percentage of 5-methylcytosine (5-mC) in hASCs genome, the MethylFlash™ Methylated DNA Quantification Kit (Epigentek) was used, following the manufacturer's instructions.

To assess the methylation levels of the *APOE* gene, bisulfite conversion of genomic DNA (500 ng each) was carried out using the EZ DNA Methylation™ Kit (Zymo Research) according to the manufacturer's protocol. PCR was carried out with 100 ng of bisulfite-converted DNA, using the Pyromark PCR Kit (Qiagen) and the primers described elsewhere [25]. PCR products were purified using streptavidin-coated sepharose beads to capture the biotin-labeled primer. Pyrosequencing was carried out on a PyroMark Q96 ID (Qiagen).

To assess mRNA levels, total RNA was isolated from exponentially growing or differentiated cells using a NucleoSpin® RNA II kit (Macherey-Nagel) according to the manufacturer's protocol. Total RNA (1 μg) was reversed-transcribed (RT) with the Transcriptor First Strand cDNA Synthesis Kit (Roche), using the manufacturer's conditions. The level of *APOE* mRNA was determined by quantitative RT-PCR (RT-qPCR) using the One-Step Real-Time system (Applied Biosytems). The expression levels were normalized using the 18 S rRNA. The ΔC_t_ method was used to calculate fold expression. StepOne software version 2.0 (Applied Biosystems) was used for data analysis.

### 2.6 Secretome analysis

Minimum media (without FBS) was collected after 48 h in contact with the cells, and then centrifuged and filtered. Protein precipitation was performed following the traditional protocol using cold acetone [26].

Aliquots were resolubilized in 0.5 M triethylammonium bicarbonate buffer (TEAB), and 50 µg of each sample was digested and labeled with iTRAQ™ labeling reagents following manufacturer's instructions (AB SCIEX, Foster City, CA) and as described in detail previously [27]. After labeling, samples were combined and concentrated under vacuum and resuspended in 0.1% ammonium formate/2% acetonitrile for tandem mass spectrometry (MS). Samples were analyzed by nano-liquid chromatography (EASY-nLC 1000, Proxeon, Thermo Scientific) coupled with an ion trap mass spectrometer (LTQ Orbitrap Velos, ThermoFisher Scientific), following protocols described elsewhere [28]. MS/MS data were processed using Protein Pilot v.4.5 software (AB SCIEX). The confidence interval for protein identification was set to ≥ 95% (p < 0.05). Only peptides with an individual ion score above the 1% False Discovery Rates (FDR) threshold were considered correctly identified. Only proteins having at least two quantifiable peptides were considered in the quantization.

For peptide mass fingerprinting, a 4800 Plus Proteomics Analyzer MALDI-TOF/TOF (Applied Biosystems) was used. Raw data file conversion tools generated mgf files, which were also searched against the human database using the Mascot Server v. 2.3.02 (AB SCIEX).

Lactate dehydrogenase (LDH) activity was determined using the commercial Lactate Dehydrogenase Colorimetric Assay Kit (Abcam®), according to the manufacturer's instructions.

### 2.7 Protein amount assessment by Western blot

Secreted proteins were concentrated using Amicon® Ultra-15 and Ultra-0.5 Centrifugal Filters (Millipore). Cells were lysed in RIPA buffer (Tris-HCl 50 mM pH = 7.4, NaCl 50 mM, sodium deoxycholate 0.5%, ethylenediaminetetraacetic acid (EDTA) 5 mM, Triton X-100 1%, protease inhibitor 1X). For Western blots, primary antibodies were against p.MT-CO1 (1:1000, 459600, Invitrogen™), SDHA (1:5000, 459200, Invitrogen™), Actin (1:2000, A2066, Sigma), APOE (1:1000, ab1906, Abcam), FN1 (1:400, ab2413, Abcam) and OXPHOS human WB antibody cocktail (Abcam, ab110411). Primary antibodies against TIM21, MRPL45, NDUFA9, p.MT-CO1, p.MT-CO2, COX4-1 and ATP5B were raised in rabbit. These antigen-antibody complexes were detected by horseradish peroxidase (HRP)-coupled secondary antibodies and enhanced chemiluminescence on X-ray films. For blue native-polyacrylamide gel electrophoresis (BN-PAGE), mitochondria were solubilized in buffer (1% digitonin, 20 mM Tris-HCl, pH 7.4, 0.1 mM EDTA, 50 mM NaCl, 10% (w/v) glycerol, and 1 mM phenylmethylsulfonyl fluoride) to a final concentration of 2 mg/ml for 30 min at 4 °C. Lysates were cleared by centrifugation (20,000×*g*, 15 min, 4 °C) before addition of 10X loading dye (5% Coomassie brilliant blue G-250, 500 mM 6-aminohexanoic acid, and 100 mM Bis-Tris, pH 7.0) and separated on 4–14% polyacrylamide gradient gels as described [29].

### 2.8 Statistics

The statistical package StatView 6.0 was used to perform all the statistics. Data for mean and standard deviation are presented. The Kolmogorov–Smirnov test was used to check the normal distribution. The unpaired two-tailed *t*-test was used to compare parameters. P values lower than 0.05 were considered statistically significant.

## 3 Results

### 3.1 Differences between human adipose tissue-derived stem cell and adipocyte secretomes

In this study, we used hASCs from two different individuals (hASCs-1 and 2). Both proliferative and differentiating hASCs were simultaneously cultured. After three weeks growing in differentiation media, approximately half of the hASCs showed adipogenic conversion [7]. Then, proliferation and differentiation media were removed and both cell cultures, hASCs and adipocytes, were 3-times washed with PBS and incubated for 2 additional days in a serum-free Dulbecco's modified Eagle's medium (DMEM) synthetic media. The secretome analysis displayed differences in the concentration of 31 proteins between media from proliferative and differentiated cells (Table S1). Eight of them were found different in only one of the differentiation processes (hASCs-1 or 2), while 23 were found different in both of them (hASCs-1 and 2).

Besides of secreted proteins, these media could contain leaked proteins. To rule out cell lysis, we determined the cytosolic enzyme activity LDH in the culture medium and normalized it by the number of cells. This activity was compared to that of a positive control obtained by cell lysis (freeze-thawing). Normalized LDH activity was only a small percentage [12.1% ± 6.1 (8), P < 0.0001] of that from the positive control [100% ± 3.1 (3)], meaning that very low cell lysis occurred during the 2 days culture period. On the other hand, keratins are usually considered contaminants coming from users’ skin, hair and nails [30]. Moreover, according to the human protein reference database (HPRD, http://www.hprd.org/), of all these proteins, only keratins and fatty acid-binding protein 4 (FABP4) lacked the secretory signal peptide. However, FABP4 is secreted through a non-classical pathway [31]. Excepting keratins, all these proteins had been previously reported in other studies on adipocyte secretomes [32]. Therefore, all these results suggested that these proteins were truly differentially secreted by adipocytes.

During adipocyte differentiation, secretion of 21 and 10 of these proteins was down and up regulated, respectively. Most of them were related with the extracellular matrix. Surprisingly, we did not find adiponectin and leptin in the secretome analysis despite that, using enzyme-linked immunosorbent assay (ELISA), we had previously observed a 4- and 6-times increase, respectively, in their culture medium levels [7]. However, leptin is mainly detected with antibody-based methods and its absence is not a rare observation for this kind of analysis [33].

### 3.2 Linezolid modifies the adipocyte secretome

LIN is a ribosomal antibiotic initially developed to treat Gram-positive bacterial infections. Moreover, the latest World Health Organization (WHO) guidelines have included linezolid in the list of core second line agents to treat multidrug resistant tuberculosis [16]. The LIN dose used in clinical practice is 600 mg every 12 h. The steady-state peak serum concentrations (44.5–80.0 μM) are reached 0.5–2 h after oral administration [17].

**Fig. 1:**
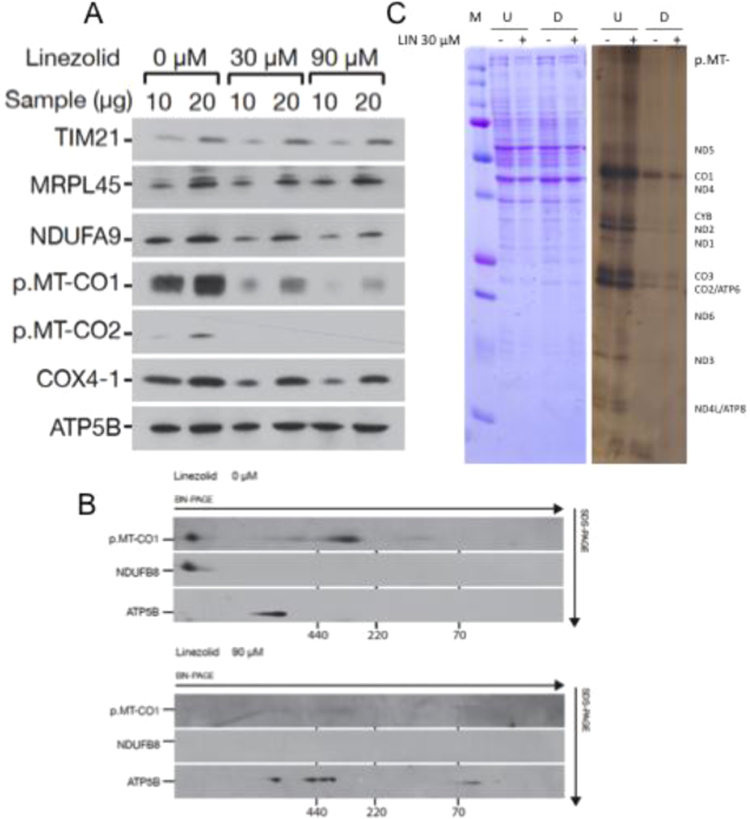
Linezolid effect on OXPHOS complexes. A) hASCs mitochondrial protein levels. Immunoblot images for different mitochondrial proteins. B) Levels of hASCs OXPHOS complexes. Image of a BN/SDS-PAGE gel. C) Adipocyte mitochondrial protein synthesis. Representative image of gels showing the electrophoretic patterns of mitochondrial translation products (right panel) and loading control (left panel). M, U, D, - and + code for molecular weight marker, undifferentiated and adipocyte-differentiated hASCs and 30 μM linezolid unexposed or exposed, respectively.

We had previously observed that 30 μM LIN decreased hASCs mitochondrial protein synthesis [7]. mtDNA only codes OXPHOS polypeptides. To confirm the LIN effect on OXPHOS function, we studied the steady-state levels of different hASCs nuclear DNA (nDNA)- and mtDNA-encoded proteins. LIN (30 μM and 90 μM) does not alter the levels of nDNA-encoded ATP5B (a CV subunit), MRPL45 (a mitochondrial ribosomal protein) and TIM21 (a presequence translocase subunit from the mitochondrial inner membrane) mitochondrial proteins (Fig. 1A). However, these LIN concentrations decrease the levels of ETC complexes subunits [Complex I (CI)- NDUFA9; Complex IV (CIV)- p.MT-CO1, 2 and COX4-1], despite some of them (NDUFA9 and COX4-1) are nDNA-encoded proteins (Fig. 1A). As mtDNA-encoded polypeptides are important for complexes assembly [34], a reduction in their levels could secondarily decrease nDNA-encoded ETC proteins. Interestingly, a two-dimension electrophoresis of samples from 90 μM LIN-treated hASCs (BN/SDS-PAGE) showed CI and CIV vanishing and that ATP5B was mainly associated to CV sub-complexes (Fig. 1B). These sub-complexes usually appear when mtDNA-encoded p.MT-ATP6 or 8 subunit levels are very low [35]. 30 μM LIN also decreases mitochondrial protein synthesis in hASC-derived adipocytes (Fig. 1C). Thus, the LIN inhibition of mitochondrial translation is responsible for the reduction in adipocyte CIV activity and quantity [7].

**Fig. 2:**
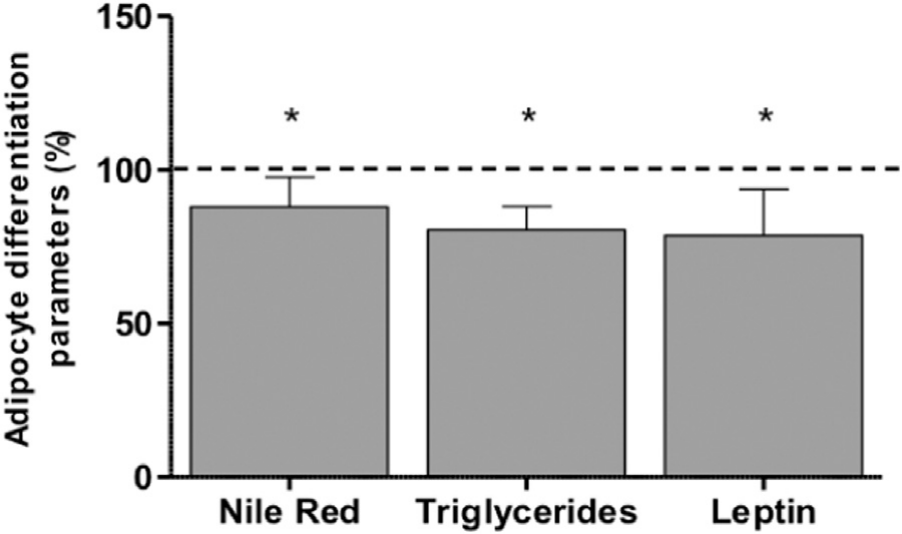
Levels of different adipocyte differentiation parameters in 30 μM linezolid-treated cells. Dashed line (100%) represents the mean values for untreated cells. The bars indicate the percentage of the different adipocyte variables. Error bars represent the standard deviation. *, P ≤ 0.0002 (versus untreated cells).

This effect on mitochondrial parameters was accompanied by a reduction in adipocyte TGs and secreted leptin [7]. These studies were performed in two different hASCs obtained from a commercial company (no phenotypic information is provided by the company). To avoid potential biases from unknown donors, we extended our analysis to encompass 11 other hASCs from known donors (Table S2). We verified that 30 μM LIN significantly decreased Nile Red lipophilic staining, intracellular TGs levels and secreted leptin (Fig. 2).

**Fig. 3:**
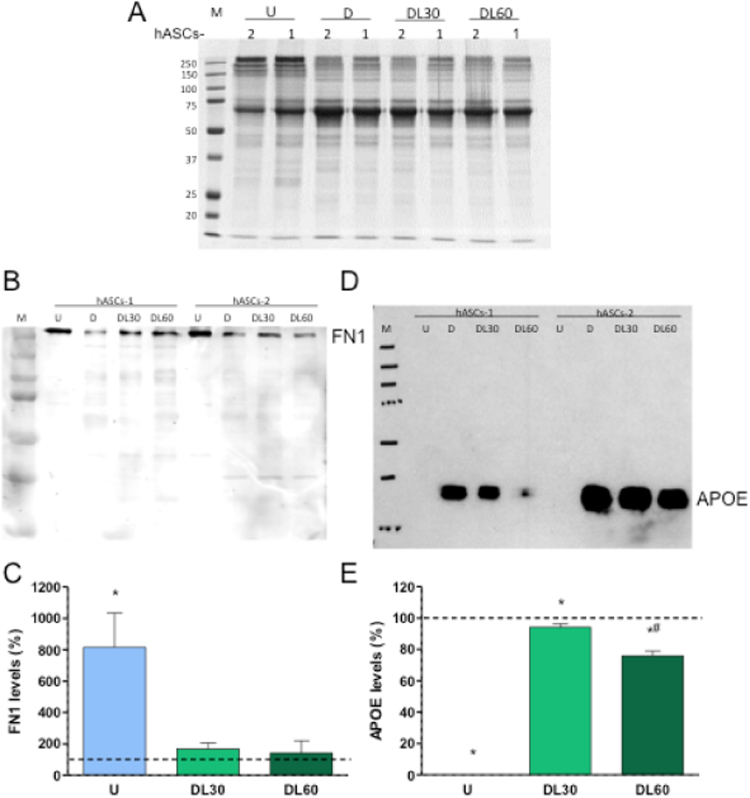
Linezolid effect on secreted fibronectin (FN1) and apolipoprotein E (APOE) levels. A) Protein loading control. Image of a SDS-PAGE gel for adipocyte-differentiated human adipose tissue-derived stem cells (hASCs) secretome samples after Coomassie blue staining. B) FN1 levels. Immunoblot image from adipocyte-differentiated hASCs exposed to different linezolid concentrations. C) Graph representing fibronectin relative quantity. D) APOE levels. Immunoblot image from adipocyte-differentiated hASCs exposed to different linezolid concentrations. As we were particularly interested in the APOE protein, and it had been reported that the specificity of many antibodies to its target protein is very low [63], we verified its specificity by performing peptide mass fingerprinting of the band obtained from the Western blot analysis of the culture medium. APOE was one of the two detected proteins in this band (Table S4). E). Graph representing APOE quantity. M, U, D, DL30 and DL60 code for molecular weight marker, undifferentiated hASCs, 0, 30 and 60 μM linezolid-exposed adipocyte-differentiated hASCs, respectively. Dashed line (100%) represents the mean values for linezolid-untreated adipocyte differentiated hASCs. The bars indicate the protein percentage of the undifferentiated and linezolid-treated adipocyte-differentiated hASCs. Error bars represent the standard deviation. *, P ≤ 0.0433 (versus linezolid-untreated adipocyte differentiated hASCs). #, P = 001 (versus 30 μM linezolid-treated adipocyte-differentiated hASCs).

The culture in the presence of 0, 30 or 60 μM LIN changed the concentration of 13 proteins, including 4 keratins, in only one of the adipocyte differentiation processes (Table S3). For 5 and 8 proteins, LIN decreased and increased, respectively, their secretion. Four of them, FABP4, apolipoprotein E (APOE), plasminogen activator inhibitor 1 (PAI-1) and complement factor D (adipsin) were not related with cytoskeleton or extracellular matrix. FABP4 and APOE levels rose along differentiation, but LIN softened this increase. Adipsin amount elevated during differentiation and LIN potentiated this upsurge. PAI-1 quantity decreased in the adipocyte differentiation, but LIN tempered this drop. At 60 μM LIN, the difference in secretion was close to be significant in both adipocyte differentiation processes for two proteins, Fibronectin (FN1) and APOE. To confirm this result, we analyzed these FN1 and APOE proteins by Western blot. There was a significant decrease in secreted FN1 levels after adipocyte differentiation. This reduction was a little mitigated when differentiation was performed in the presence of 30 or 60 μM LIN (Fig. 3A-C). On the other hand, there was not signal for APOE protein in hASCs. 30 or 60 μM LIN significantly diminished the increase in secreted APOE levels after adipocyte differentiation (Fig. 3A, D, E).

### 3.3 Linezolid alters global DNA methylation

**Fig. 4:**
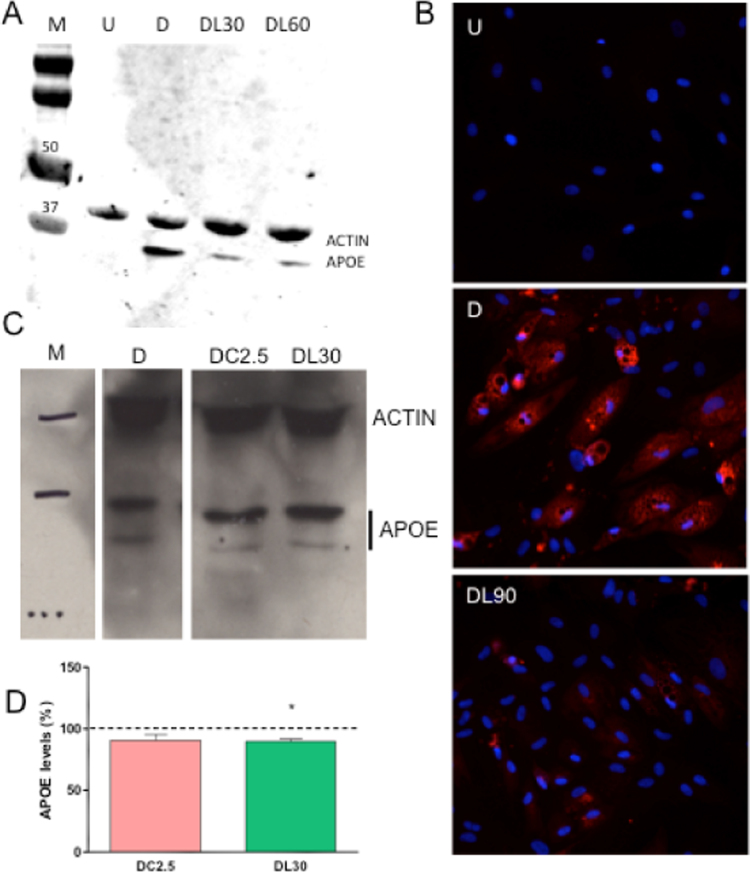
Linezolid effect on intracellular apolipoprotein E (APOE) levels. A) APOE levels. Immunoblot image from adipocyte-differentiated human adipose tissue-derived stem cells (hASCs) exposed to different linezolid concentrations. Actin levels are used as a loading control. B) Immunofluorescent staining of adipocyte-differentiated hASCs. Representative image of an APOE immunocytochemical analysis. C) APOE levels of adipocyte-differentiated hASCs treated with ribosomal antibiotics. Representative images of immunoblots for APOE and actin. D) Graph representing APOE quantity. DC2.5 codes for 2.5 μM cloramphenicol-exposed adipocyte-differentiated hASCs. M, U, D, DL30, DL60 and DL90 code for molecular weight marker, undifferentiated hASCs, 0, 30, 60 and 90 μM linezolid-exposed adipocyte-differentiated hASCs, respectively. Dashed line (100%) represents the mean values for adipocyte-differentiated hASCs. The bars indicate the APOE percentage of the chloramphenicol or linezolid-treated adipocyte-differentiated hASCs. Error bars represent the standard deviation. *, P = 0.0273 (versus untreated-adipocyte differentiated hASCs).

Next, we determined APOE levels in cell homogenates. APOE protein was found in adipocytes but it was not present in hASCs (Fig. 4A). LIN (30 and 60 μM)-treated adipocytes showed lower APOE amount. Immunocytochemical analysis of cells treated with 90 μM LIN confirmed the effect of this antibiotic on intracellular APOE quantity (Fig. 4B). Other mitochondrial translation inhibitor, CAM, at concentration (2.5 μM) well below the lower limit of its therapeutic range (46.4–92.8 μM) [36], decreased adipocyte TGs and secreted leptin [7]. This drug also tends to decrease APOE protein levels (Fig. 4C, D).

**Fig. 5:**
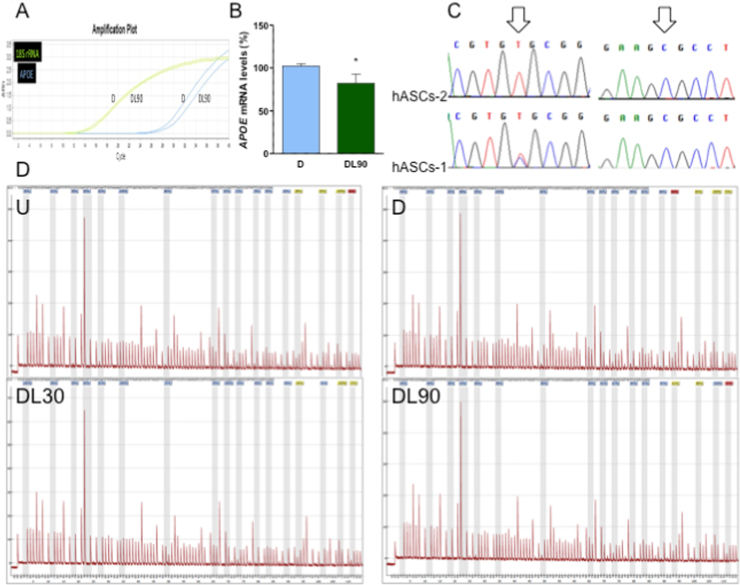
Apolipoprotein E (APOE) expression. A) Representative image of a RT-qPCR result. B) Graph representing *APOE* mRNA levels, normalized using the 18S rRNA, in adipocyte-differentiated human adipose tissue-derived stem cells (hASCs). C) *APOE* genotype. D) *APOE* gene CGI methylation. U, D, DL30 and DL90 code for undifferentiated hASCs, 0, 30 and 90 μM linezolid-treated adipocyte-differentiated hASCs, respectively. *, P = 0.0322 (versus untreated-adipocyte differentiated hASCs).

To determine the reasons for this decrease in cell APOE levels, we measured *APOE* mRNA expression by RT-qPCR. The mRNA levels were decreased in 90 μM LIN-treated adipocytes (Fig. 5A, B). *APOE* gene contains a CpG island (CGI) with transcriptional enhancer/silencer activity in exon 4. *APOE2* and *APOE4* alleles reduce and increase, respectively, one CpG dinucleotide when compared with *APOE3* allele. Therefore, the *APOE* allele could alter the methylation landscape and the gene transcription [25]. The hASCs-1 and hASCs-2 genotypes were *APOE3/4* and *APOE3/3*, respectively (Fig. 5C). These are the most frequent genotypes in the Spanish population (16.5% and 72.0%, respectively) [24], and are also the most common almost everywhere [37]. We determined this CGI methylation state. We did not find differences in methylation levels of 2 different regions from *APOE* exon 4 among hASCs and adipocytes or among LIN-treated and untreated adipocytes (Fig. 5D).

**Fig. 6:**
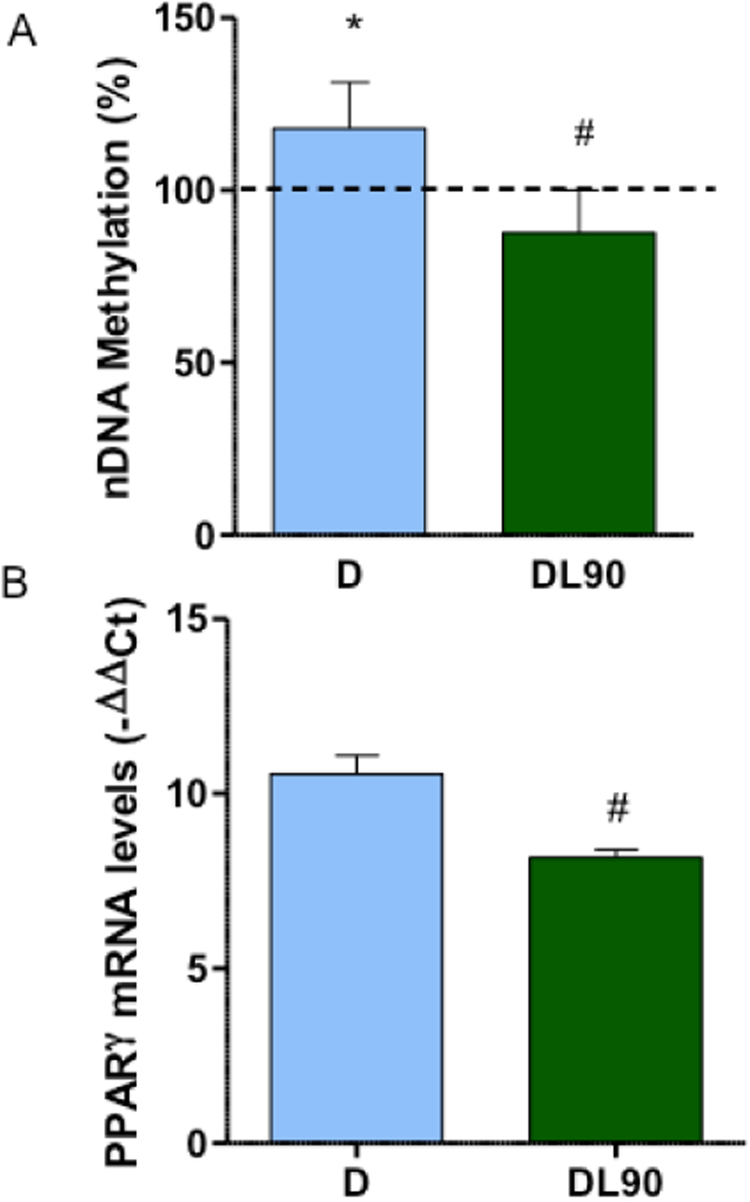
Nuclear DNA methylation and *PPARγ* expression. A) Global nDNA methylation of untreated or linezolid-treated adipocyte-differentiated human adipose tissue-derived stem cells (hASCs). B) *PPARγ* mRNA levels, normalized using the 18S rRNA, of untreated or linezolid-treated adipocyte-differentiated hASCs. Dashed line (100%) represents the mean values for hASCs. The bars indicate the percentage in unexposed or 90 μM linezolid-exposed adipocyte-differentiated hASCs. Error bars represent the standard deviation. *, P = 0.015 (versus hASCs). #, P ≤ 0.0148 (versus untreated-adipocyte differentiated hASCs).

It has been published that 5-aza-2′-deoxycytidine (5azadC), a reagent having inhibitory effects on DNA methyltransferase, reduced adipocyte differentiation of mouse 3T3-L1 cells [38] and hMSCs [39]. To check if LIN effect on adipocyte differentiation is mediated through nDNA methylation, we analyzed this variable in unexposed or LIN-exposed adipocytes differentiated hASCs. Adipocyte differentiation provoked a significant increase in global nDNA methylation (Fig. 6A). However, this increase was avoided when cells were treated with 90 μM LIN.

It was reported that 5azadC decreased peroxisome proliferator-activated receptor gamma (*PPARγ*) expression, an important factor for adipocyte differentiation [39], [40]. It has been recently found that NRTIs altered nDNA methylation [41], and downregulated *PPARγ* and mtDNA-encoded genes expression [42]. Moreover, ethidium bromide, a DNA intercalating agent that provokes mtDNA depletion, reduced 3T3-L1 *MT-CO1* and *PPARγ* mRNA levels [4]. To confirm an effect on *PPARγ* expression of OXPHOS xenobiotics, we determined the *PPARγ* mRNA level after LIN exposure and observed that LIN (90 μM) significantly decreased *PPARγ* mRNA amount (Fig. 6B).

**Fig. 7:**
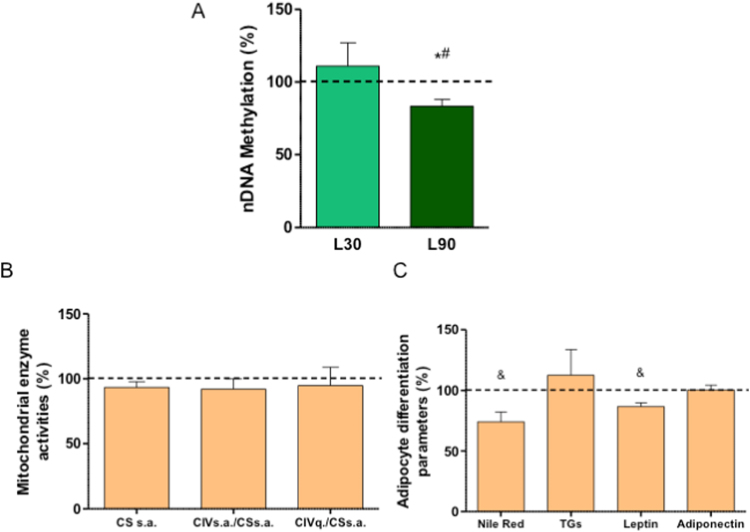
Linezolid long-term effects. A) Global nDNA methylation of untreated (dashed line) or 30 and 90 μM linezolid (L30 and L90)-treated human adipose tissue-derived stem cells (hASCs). B) Mitochondrial enzymes. CSsa, CIVsa and CIVq code for citrate synthase and respiratory complex IV specific activities and respiratory complex IV quantity, respectively. C) Parameters of adipocyte differentiation. Dashed line (100%), in B and C, represents the mean values for LIN-untreated adipocyte differentiated hASCs. The bars indicate the percentage in adipocyte-differentiated hASCs previously treated with linezolid 30 μM. Error bars represent the standard deviation. *, P = 0.0194 (versus untreated hASCs). #, P = 0.048 (versus 30 μM linezolid-treated hASCs). &, P ≤ 0.0367 (versus LIN-untreated adipocyte differentiated hASCs).

The LIN toxicity limits the treatment time to 28 days, the Food and Drug Administration (FDA)-approved maximum duration [16]. Thus, the differentiation of LIN-exposed adipocytes during this period could be altered. Fortunately, most toxic effects are reversible and corrected by discontinuing LIN therapy [16]. However, as LIN exposure during differentiation affects nDNA methylation, to know if LIN might have a future effect on hASCs differentiation to adipocyte, we looked for potential hASCs epigenetic effects and analyzed the global nDNA methylation. Thus, this process was also diminished in 90 μM LIN-treated hASCs (Fig. 7A). Interestingly, when hASCs were treated with 30 μM LIN for 10 days and then differentiated in the absence of LIN, they were able to recover CIV activity and quantity but adipocyte differentiation was affected and Nile Red staining and secreted leptin was diminished (Fig. 7B, C).

## 4 Discussion

The number and set of differentially secreted proteins between preadipocytes and adipocytes varies in the different reports on adipocyte secretomes, despite a large percentage of cells differentiate to adipocytes in all of them. Maybe the species from which the cell type was obtained, the cell type, differentiation protocol and method of analysis are responsible for these large variations. However, some proteins are frequently found in many of these reports [32]. Thus, some of them are related with remodeling of the extracellular matrix, a crucial event during adipocyte differentiation, in which cell morphology converts from a fibroblast-like to a spherical shape [43], and some others are adipokines, a variety of circulating factors, which regulate systemic metabolism.

We had previously observed that LIN, an inhibitor of the mitochondrial protein synthesis, was able to decrease adipocyte differentiation and leptin secretion [7]. Here, we report a contrary effect of LIN on secreted APOE and FN1 protein levels. LIN also affects some adipokines, such as FABP4, PAI-1 and Adipsin.

The amount of FN1 protein decreases during adipocyte differentiation but LIN tends to mitigate this decline. The effect on FN1 secretion is different in other cells with OXPHOS dysfunction. Thus, levels of this protein were decreased in the extracellular space of rho^0^ (without mtDNA), mutant (mtDNA-deleted), or CI-inhibited (rotenone-treated) osteosarcoma 143B cells. This decrease was not detected in rotenone-treated rho^0^ cells, proving this to be an effect dependent on inhibition of functional mitochondria [44]. Tumoral osteosarcoma 143B cells are proliferative and undifferentiated cells. Therefore, OXPHOS dysfunction can have different secretory effects on different cell types.

It was observed that frozen sections of human white adipose tissue lack of FN1 immunoreactivities, but the same sections showed positive staining around blood vessels, nerves and mammary glandular tissue. Thus, the absence of FN1 in the subcutaneous adipose tissue is an important prerequisite for the maintenance of the normal function and morphology of the fully differentiated adipocytes [45]. On the other side, it has also been informed that adipocyte differentiation of mouse 3T3-F442A cells is inhibited by FN1 [46]. Thymidine analog NRTIs provoke mtDNA depletion and OXPHOS dysfunction. Some patients receiving this medication can present facial and limb lipoatrophy [11]. OXPHOS dysfunction in these NRTIs-treated patients perhaps avoids large decreases in FN1 secretion and higher extracellular FN1 levels would inhibit adipocyte differentiation.

Similar to other reports, we have observed that secreted APOE levels increase with adipocyte differentiation of hASCs. We have also noted that OXPHOS xenobiotic LIN tends to moderate this rise. The increase in the amount of secreted FN1 protein and other adipocyte factors suggests that LIN has no effect on the general secretion process. This fact is confirmed by the reduction in the intracellular APOE protein and mRNA amount.

Other observations also support OXPHOS as a regulatory factor for *APOE* expression. Thus, the intracellular free cholesterol content regulated *APOE* mRNA expression in 3T3-L1 adipocytes [47]. The cholesterol metabolites oxysterols interact with the liver receptor X (LXR) and regulate *APOE* expression in adipose tissue [48]. In 3T3-L1 adipocytes, PPARγ agonists increased, and tumor necrosis factor alpha (TNFα) decreased, LXR binding to *APOE* gene and the APOE secretion [49]. The TNFα effect was mediated by NF-κB binding to *APOE* gene [50]. Reactive oxygen species (ROS) also reduced adipocyte *APOE* expression via the NF-κB pathway [51]. Very interestingly, PPARγ agonists induce adipose mitochondrial biogenesis and increase oxygen consumption in mouse 3T3-L1 and C3H/10T1/2 cells [52]. 3T3-L1 adipocytes treated with TNFα decreased mitochondrial biogenesis, mtDNA-encoded gene expression, oxygen consumption and mitochondrial inner membrane potential [53]. TNFα also increased ROS levels, because these compound usually increase with OXPHOS dysfunction. Thus, along with the previous observations, the LIN effect on OXPHOS function might explain the reduction of cellular and secreted APOE protein amount. Moreover, we previously observed that ribosomal antibiotics CAM and LIN tent to increase ROS levels [7]. Therefore, these drugs would also act through the NF-κB pathway.

We have also found that adipocyte differentiation of hASCs is accompanied by an increase in global nDNA methylation. It was previously reported that inhibiting nDNA methylation in 3T3-L1 preadipocytes, or hMSCs, by 5azadC significantly inhibited adipogenesis [38], [39], [40]. Thus, demethylating and upregulating *WNT10A* gene expression, which codes for a key factor to determine the fate of hMSCs, provoke a decreased *PPARγ* expression and reduced adipocyte differentiation [39], [40]. LIN, inhibiting mitochondrial translation, is able to affect nDNA methylation and reduce *PPARγ* mRNA levels. Similarly, it was reported that rho^0^ cells showed significant changes in the methylation pattern of a number of genes. These methylation changes were reversed by the restoration of mtDNA in these cells [54]. Mice harboring a dysfunctional POLG, the enzyme involved in mtDNA replication, displayed altered nDNA methylation [55]. Also, nDNA methylation levels were different in transmitochondrial cell lines (cybrids) harboring different mtDNAs [56], [57]. As cybrids share the same nDNA and culture conditions, this result meant that mtDNA genetic variation altered OXPHOS function and caused retrograde signals that modified nDNA methylation. Very interestingly, it has been recently found that cybrids from a particular mtDNA genetic background showed higher levels of nDNA methylation and *APOE* transcription than those from other mtDNA haplogroup [58]. All these results suggest that OXPHOS function, modifying nDNA, can affect PPARγ expression, APOE production and adipocyte differentiation.

LIN exposure provokes similar effects on adipocyte secretion to those caused by knockout *APOE* gene. Thus, FN1 protein levels were increased in *APOE*^-/-^ mice. APOE interrupts a mechanically driven feed-forward loop that increases the expression of FN1 [59]. Plasma levels of PAI-1 in *APOE*^-/-^ mice were significantly higher than that of controls [60]. Mice with selective suppression of adipose tissue APOE expression and normal circulating APOE levels had a lower leptin, and a higher adiponectin, expression [61]. In humans, APOE knockdown hMSC-Tert adipocytes strongly decreased *FABP4* gene expression levels [62]. These facts suggest that LIN effect on adipokine secretion is APOE mediated.

## 5 Conclusions

Irrespective of the side effects on other tissues, affecting adipocyte APOE production and, secondarily, other adipokines, LIN can also contribute to systemic effects. In fact, it has been reported that selective suppression of adipose *APOE* expression impacts systemic metabolic phenotype and adipose tissue inflammation [61]. These results suggest that OXPHOS xenobiotics acting on adipocytes alter APOE and adipokine production, thus contributing to their systemic effects.
