# Supplementary material for: Pharmacologic concentrations of linezolid modify oxidative phosphorylation function and adipocyte secretome
Source: Redox Biol. 2017 May 31;13:244–54. doi: 10.1016/j.redox.2017.05.026 (PMC5466587; doi:10.1016/j.redox.2017.05.026)
Supplement: Supplementary file 1 — Supplementary material [file mmc1.doc]

| **Gene** | **Protein** | **Accession Code** | **Diff / Undiff** | **Signal Peptide** | **Location** |
| --- | --- | --- | --- | --- | --- |
| *POSTN* | Periostin | Q15063 | 0.17 | Y | E |
| *TGFB1* | Transforming growth factor-beta-induced protein ig-h3 | Q15582 | 0.18 | Y | E |
| *MMP2* | 72 kDa type IV collagenase | P08253 | 0.24 | Y | E |
| *COL1A* | Collagen alpha-1 (I) chain | P02452 | 0.24 | Y | E |
| *FN1* | Fibronectin | P02751 | 0.25 | Y | E |
| *FBN1* | Fibrillin-1 | P35555 | 0.27 | Y | E |
| *COL1A2* | Collagen alpha-2 (I) chain | P08123 | 0.29 | Y | E |
| *SERPINE1* | Plasminogen activator inhibitor 1 | P05121 | 0.31 | Y | E |
| *COL6A3* | Collagen alpha-3 (VI) chain | P12111 | 0.31 | Y | E |
| *COL3A1* | Collagen alpha-1 (III) chain | P02461 | 0.32 | Y | E |
| *OLFML3* | Olfactomedin-like protein 3 | Q9NRN5 | 0.39 | Y | E |
| *COL6A1* | Collagen alpha-1 (VI) chain | P12109 | 0.44 | Y | E |
| *COL5A1* | Collagen alpha-1 (V) chain | P20908 | 0.44 | Y | E |
| *KRT10* | Keratin, type I cytoskeletal 10 | P13645 | 0.48 | N | C |
| *KRT1* | Keratin, type II cytoskeletal 1 | P04264 | 0.51 | N | M |
| *KRT9* | Keratin, type I cytoskeletal 9 | P35527 | 0.52 | N | C |
| *KRT2* | Keratin, type II cytoskeletal 2 | P35908 | 0.54 | N | C |
| *TF* | Serotransferrin | P02787 | 0.54 | Y | E |
| *C1S* | Complement C1s subcomponent | P09871 | 0.57 | Y | E |
| *COL5A2* | Collagen alpha-2 (V) chain | P05997 | 0.57 | Y | E |
| *LAMC1* | Laminin subunit gamma-1 | P11047 | 0.59 | Y | E |
| *NID1* | Nidogen-1 | P14543 | 1.76 | Y | E |
| *THBS1* | Thrombospondin-1 | P07996 | 1.77 | Y | E |
| *SERPINF1* | Pigment epithelium-derived factor | P36955 | 2.08 | Y | E |
| *GSN* | Gelsolin | P06396 | 2.23 | Y | E |
| *COL4A2* | Collagen alpha-2 (IV) chain | P08572 | 2.57 | Y | E |
| *FABP4* | Fatty acid-binding protein, adipocyte | P15090 | 3.30 | N | C |
| *TNC* | Tenascin | P24821 | 3.61 | Y | E |
| *CFD* | Complement factor D | P00746 | 4.12 | Y | E |
| *APOE* | Apolipoprotein E | P02649 | 4.20 | Y | E |
| *COMP* | Cartilage oligomeric matrix protein | P49747 | 4.73 | Y | E |

Table S1- List of proteins differentially secreted between human adipose tissue-derived stem cells (hASCs) and adipocytes. Diff / Undiff, Y, N, E, C and M code for differentiated / undifferentiated, yes, no, extracellular, cytoplasmic and plasma membrane, respectively. Gray and white lines indicate proteins differentially secreted in only one (hASCs-1 or 2) or both (hASCs-1 and 2) differentiation processes, respectively. Cutoffs to consider over- and under-expressed proteins were 1.3 and 0.7 ratios, respectively.

| **Sample** | **Sex** | **Age**  **(years)** | **BMI**  **(kg/m^2^)** | **Pathology** | ***APOE* genotype** |
| --- | --- | --- | --- | --- | --- |
| 9 | F | 75 | 29.2 | AHT | *APOE3 / APOE3* |
| 10 | F | 42 | 23.4 | - | *APOE3 / APOE3* |
| 12 | F | 60 | 24.2 | - | *APOE3 / APOE4* |
| 15 | F | 41 | 20.0 | - | *APOE3 / APOE3* |
| 16 | F | 77 | 41.1 | AHT / Dyslipidemia | *APOE3 / APOE3* |
| 24 | F | 53 | 45.4 | - | *APOE3 / APOE3* |
| 25 | F | 55 | 43.5 | AHT | *APOE3 / APOE3* |
| 31 | F | 32 | 57.7 | - | *APOE3 / APOE3* |
| M51 | F | 51 | - | - | - |
| V85 | M | 85 | - | - | - |
| hASC-3 | - | - | - | - | *APOE3 / APOE3* |

Table S2. Phenotype features of human adipose tissue-derived stem cells (hASCs) donors. BMI, F, M AHT code for body mass index, female, male and arterial hypertension, respectively.

| **Gene** | **Protein** | **Accession Code** | **L30 Diff / Diff** | **L60 Diff / Diff** | **L60 Diff / L30 Diff** |
| --- | --- | --- | --- | --- | --- |
| *FABP4* | Fatty acid-binding protein, adipocyte | P15090 | - | - | 0.68 |
| *KRT9* | Keratin, type I cytoskeletal 9 | P35527 | - | - | 0.69 |
| *APOE* | Apolipoprotein E | P02649 | 0.59 | (0.72) | 0.69 |
| *COL4A2* | Collagen alpha-2 (IV) chain | P08572 | 0.68 | - | - |
| *COL5A2* | Collagen alpha-2 (V) chain | P05997 | 0.69 | - | - |
| *FN1* | Fibronectin | P02751 | - | (1.27) | - |
| *KRT1* | Keratin, type II cytoskeletal 1 | P04264 | - | 1.37 | - |
| *KRT10* | Keratin, type I cytoskeletal 10 | P13645 | - | 1.38 | - |
| *FBN1* | Fibrillin-1 | P35555 | - | 1.49 | - |
| *MMP2* | 72 kDa type IV collagenase | P08253 | 1.35 | 1.55 | - |
| *TGFB1* | Transforming growth factor-beta-induced protein ig-h3 | Q15582 | 1.38 | 1.55 | - |
| *SERPINE1* | Plasminogen activator inhibitor 1 | P05121 | 1.42 | 1.46 | - |
| *CFD* | Complement factor D | P00746 | 1.46 | - | - |
| *KRT2* | Keratin, type II cytoskeletal 2 | P35908 | - | - | 1.55 |

Table S3. List of proteins differentially secreted after linezolid (L) exposure. Diff, L30 Diff and L60 Diff code for L-untreated, 30 μM L-treated and 60 μM L-treated differentiations, respectively. Gray and white lines indicate proteins differentially secreted in only one or both human adipose tissue-derived stem cells (hASCs) differentiation processes, respectively. Cutoffs to consider over- and under-expressed proteins were 1.3 and 0.7 ratios, respectively.

| **Protein** | **Accession**  **Code** | **MW (Da) / IP** | **MASCOT score** | **Covered**  **sequence**  **(%)** | **Paired**  **peptides**  **number** | **MS / MS**  **Data** |
| --- | --- | --- | --- | --- | --- | --- |
| APOE | P02649 | 36,246 / 5.65 | 278 | 62 | 21 | 1,033.5  LQAEAFQAR  45 |
| SPARC | P09486 | 35,465 / 4.73 | 197 | 43 | 13 | 1,497.5  AATVGSLAGQPLER  65 |

Table S4. Results of peptide mass fingerprinting for APOE identification. MW, IP and MS/MS code for molecular weight, isoelectric point and tandem mass spectrometry, respectively.
